# The feasibility and acceptability of a classroom-based physical activity program for children attending specialist schools: a mixed-methods pilot study

**DOI:** 10.1186/s12889-021-11990-4

**Published:** 2022-01-06

**Authors:** Chloe Emonson, Nicole Papadopoulos, Nicole Rinehart, Ana Mantilla, Ian Fuelscher, Lynne M. Boddy, Caterina Pesce, Jane McGillivray

**Affiliations:** 1 Deakin Child Study Centre, School of Psychology, Faculty of Health, Deakin University, Geelong, Australia; 2 School of Educational Psychology and Counselling, Faculty of Education, Monash University, Clayton, Victoria Australia; 3 Centre for Social and Early Emotional Development (SEED), School of Psychology, Faculty of Health, Deakin University, Geelong, Australia; 4 Research Institute for Sport and Exercise Sciences, Liverpool John Moores University, Liverpool, UK; 5 Department of Movement, Human and Health Sciences, University of Rome Foro Italico, Rome, Italy

**Keywords:** Primary school, Disability, Special needs, Class, Active break, Feasibility, Acceptability

## Abstract

**Background:**

Children with disabilities often engage in less than the recommended amount of daily physical activity (PA). Classroom-based PA breaks are a favourable method of promoting PA for children. However, evaluations of these programs in specialist schools are scarce, with even less research into their feasibility and acceptability. This may hinder effective implementation and program scalability. This pilot study investigated the feasibility and acceptability of implementing a classroom-based PA break program in Australian specialist school classrooms, using the Australian Joy of Moving (AJoM) program.

**Methods:**

Forty primary/junior classes and their teachers across five specialist schools implemented the AJoM program for eight weeks as the intervention group within a pilot cluster randomised controlled trial. A mixed-methods design investigated classroom teachers’ (*N* = 22; 6 males, 16 females) perspectives of the feasibility and acceptability of the program after implementation through semi-structured telephone interviews (*n* = 7 teachers), qualitative survey responses (*n* = 18 teachers) and quantitative survey items (*n* = 19 teachers). Qualitative data were analysed using predominantly deductive thematic analysis. Quantitative data were analysed using descriptive statistics.

**Results:**

Classroom-based PA breaks may be feasible for getting children with disabilities more active at school. However, considerable variation exists in teachers’ perception of the AJoM experience. While several teachers indicate that the program content could be pertinent for their class, common divergences in perceptions of feasibility and acceptability appear to relate to the age and developmental level or needs of the students in the class.

**Conclusions:**

This study provides preliminary evidence for the feasibility and acceptability of implementing classroom-based PA breaks in specialist schools. However, results demonstrate the importance of (1) allowing a high level of flexibility in the design and implementation of programs to meet the varying needs of class groups and (2) providing a large variety of resources to cater to the heterogeneity of the children.

**Trial registration:**

This trial was prospectively registered with the Australian New Zealand Clinical Trials Registry (ACTRN12619000193178) on 11 February 2019.

**Supplementary Information:**

The online version contains supplementary material available at 10.1186/s12889-021-11990-4.

## Background

The 2020 World Health Organisation guidelines recommend that all children, including those with disabilities, engage in at least an average of 60-min of moderate-to-vigorous physical activity (MVPA) per day and limit the amount of time spent engaged in sedentary behaviour [1]. Engagement in physical activity (PA) is associated with a myriad of developmental benefits in physical, emotional, individual, social, intellectual and even financial domains, as outlined in the Human Capital Model proposed by Bailey and colleagues [2]. That is, PA is a means that can contribute to “whole child” development [3]. However, many children with disabilities do not engage in sufficient levels of daily PA and spend a large amount of time in sedentary activities [4–6]. Physical inactivity increases the risk of experiencing poor health outcomes and is therefore a major public health issue [7]. Given children with disabilities are subject to high rates of adverse secondary conditions including physical (e.g., obesity) [8] and psychological (e.g., emotional and conduct problems) [9] health difficulties, Anderson and Heyne [10] demonstrate the “amplified” importance of ensuring these children experience the benefits of PA. Therefore, programs targeting the physical and psychological health of children with disabilities by increasing PA would be of benefit, possibly more so than for typically developing children.

Children with disabilities experience numerous barriers to participation in community-based PA such as unsuitable environments, low perceived levels of community supportiveness and inexperience of coaches in including children with disabilities [11–14]. This suggests that other settings may be particularly important for supporting children with disabilities to participate in PA. Indeed, these children have been shown to depend on their school setting to participate in PA more than typically developing peers [15]. For example, Einarsson et al. [15] found that children with disabilities were more active during school time compared to after school, whereas there was no statistical difference for typically developing children. Thus, the school setting is a critical venue for children with disabilities to engage in PA [16].

Majority (approximately 89%) of students with disability in Australia attend a mainstream school [17] (which enrol children with and without disability) with specialist education services and supports to assist, if required [18]. However, a small portion of children with disabilities around the world attend specialist schools, which, for the purposes of this research, refer to schools that enrol only students with disabilities or special needs [18]. For example, approximately 9% of children with special educational needs in England [19], 3% of students supported by the Individuals with Disabilities Education Act in the United States [20] and 12% of students with disability in Australia [17] attend specialist schools. In Victoria, Australia, it is up to the family to choose whether a child with disability attends a mainstream or specialist school [21]. Victorian specialist schools are specifically designed, resourced and staffed to support children with disabilities that may have high needs, with class groups generally being smaller than those in mainstream schools and formed according to children’s abilities and educational needs [18, 22].

Research suggests that children with disabilities generally spend little time at school doing MVPA, only 50% of the approximately 30 min done by typically developing peers [15]. Additionally, while research on PA engagement in Australian specialist schools is limited, Sit and colleagues [23] found that students ranging from childhood to young adulthood at specialist schools in Hong Kong spent only 4% of the school day in MVPA and 26% in light PA. MVPA was also limited during active opportunities such as Physical Education (PE; 13% of the class), recess (9%) and lunch breaks (5%) [23]. Youth (aged 6–23 years) were further found to spend 70% of the school day being sedentary [23]. Although research indicates that both children with disabilities and typically developing children spend large portions of the school day being sedentary, it may be particularly necessary to increase PA opportunities at school for children with disabilities, given they participate in less PA at school compared to typically developing peers despite being more reliant on the school setting to accrue PA [15]. Moreover, low amounts of PA and high amounts of sedentary behaviour appear to have independent negative effects on a range of health outcomes for all individuals [24], although, this is an issue of “amplified importance” for children with disabilities [10]. Given children spend much of their waking time at school, the provision of additional school-based PA programs may be valuable in strengthening attempts to increase PA engagement for children with disabilities [25–27], which may assist in reducing adverse health effects.

A promising method of increasing in-school PA is through the use of classroom-based PA. Classrooms are well-placed to support PA participation given the considerable portion of time students spend in this setting [28]. Moreover, classroom-based PA fits within State and national initiatives around the world to increase children’s PA [29, 30]. Notably, classroom-based PA programs not only attempt to increase PA but can also interrupt prolonged sitting time [27]. They have been widely implemented in mainstream schools and have shown to contribute to several benefits including health- (e.g., for PA levels) and academic-related (e.g., for classroom behaviour and academic achievement) benefits (e.g., [31–34]). As such, literature suggests that classroom-based PA should be viewed by schools as best practice [35]. Classroom-based PA can be implemented in various forms including active lessons (i.e., integrating PA into academic content) or active breaks (e.g., brief stand-alone PA sessions between or during academic lessons) [28].

Brief classroom-based PA breaks (e.g., 5–10 min; often referred to as ‘active breaks’ or ‘movement breaks’) are particularly attractive to teachers given the time constraints they often operate under [36], and have the potential to increase children’s PA in both mainstream and specialist schools [27, 34]. Literature demonstrates the feasibility and acceptability of implementing PA breaks in mainstream classrooms (e.g., [37, 38]). Notably, McMinn et al. [39] and Mazzoli et al. [27] demonstrate the potential for classroom-based PA breaks to also be used with children with disabilities and special educational needs. However, literature identifies differences between mainstream and specialist schools that indicate why classroom-based PA breaks implemented in mainstream schools may not necessarily transfer to specialist schools. For example, specialist schools utilise specialised, intensive instruction [40] and have significant heterogeneity between students [41]. Differences in environments and developmental age of students may also influence the implementation of classroom-based PA breaks. Indeed, McMinn et al. [39] and Mazzoli et al. [27] identified factors to be considered before implementing classroom-based PA breaks with children with disabilities and special educational needs including their various needs (e.g., in relation to cognitive functioning), level of physical and emotional development, and potential apprehension towards new activities. These considerations align with the postulates of the Universal Design for Learning (UDL) framework, which state that no two students are the same and that learning should be tailored to individual students [42].

It is therefore not appropriate to directly apply what is known about classroom-based PA breaks in mainstream schools to specialist schools. This demonstrates the importance of understanding the landscape of classroom PA in specialist schools separately. A systematic mapping review of class time PA programs that have been implemented in specialist school settings revealed that only 11 out of 34 programs identified had been implemented through short (≤ 20 min) sessions and only seven of these were delivered by the classroom teacher [43]. Additionally, the review found that class time PA programs in specialist schools of *any* length had not been extensively evaluated, identifying only 23 programs involving a PA component that had been evaluated, four of which were implemented through brief sessions by the classroom teacher [43], representing an active break. Thus, further evaluation of PA break programs in specialist school classrooms is required to advance the limited evidence base and inform classroom practice.

Moreover, to the best of our knowledge, only one study has specifically investigated the feasibility of an active break in specialist schools [38]. That is, while studies have evaluated outcomes associated with children participating in brief class time PA sessions in specialist classes (see [43]), Mazzoli and colleagues [38] were the first to conduct a feasibility study of an active break in a specialist school. The active break consisted of a cognitively challenging motor task, whose feasibility seems lower in specialist compared to mainstream schools, but may increase if motor tasks are tailored to children’s specific needs [38]. Understanding whether a practice is feasible plays an important role in scaling-up program implementation for widespread adoption in special education [44], and is therefore vital to progressing this field of research. Additionally, the implementation and success of classroom-based PA programs is significantly influenced by the decisions of classroom teachers [35]. Thus, limited research into the feasibility and acceptability of these activities from the teachers’ perspective in specialist schools is a considerable gap in current literature.

To summarise, (a) literature describes factors to be considered before implementing classroom-based PA breaks in specialist schools including children’s physical and emotional development and apprehension towards new activities [39], (b) the implementation and evaluation of classroom-based PA breaks in specialist schools to date is limited, and (c) feasibility evaluations are particularly lacking, hindering the ability to understand whether programs are scalable. Given this, the current research evaluates the feasibility and acceptability of implementing a classroom-based PA break program in specialist schools. Specifically, the Australian Joy of Moving (AJoM) program will be implemented, as it is a novel and psychologically-focussed classroom-based PA break program containing elements aligned with the implementation considerations identified by McMinn and colleagues [39]. For example, the AJoM program contains psychoeducation emphasising the benefit of PA for psychological wellbeing using storybooks, which provide a visual support that could assist with students’ anxiety and apprehension of transitioning to a new activity [45, 46]. The program also takes a flexible approach to implementing movement activities to allow tailoring to the developmental abilities of the class.

The aim of this pilot study is to investigate the feasibility and acceptability of implementing a classroom-based PA break program in Australian specialist primary/junior school classrooms (which consist of students approximately 5–12 years of age). This research is conducted alongside a trial of the AJoM program in mainstream primary schools after undergoing some adaptation. Since literature indicates that classroom-based PA break programs developed for mainstream schools may not directly transfer to specialist schools, a distinct evaluation of feasibility and acceptability is warranted. Additionally, this research may inform considerations required for the future use of these programs and subsequent efficacy and effectiveness studies necessary to scale classroom-based PA break programs with children attending specialist schools.

## Methods

### Study design and setting

Although this research was part of a larger pilot cluster randomised controlled trial, the study reported here used a mixed-methods design to investigate teachers’ experience with the program post-implementation and therefore reports data from the intervention group only. Thirty-four specialist schools in Victoria, Australia were invited to take part in the study. Nine of these schools (catering for students aged between 5 and 18 years) provided principal consent. Five were allocated to receive the AJoM program and form the settings for this study. These specialist schools primarily cater for students with mild to profound intellectual disabilities. While, collectively, the schools cover the full range of intellectual disability, each school has its own eligibility criteria regarding severity. Although schools had both primary/junior and secondary/senior level classes, only the primary/junior classes were eligible for the study, as the AJoM program was not designed for secondary school students. There were 55 primary/junior classes across the five participating schools.

### Participants

In an attempt to attain the largest possible sample size to understand the practicalities of implementing classroom-based PA breaks in diverse specialist schools, all primary/junior classroom teachers at consenting schools were invited to participate, and no minimum enrolment criterion per school was imposed. Classes often had educational support staff in addition to the classroom teacher. However, given the presence of education support staff may have been inconsistent across each class at participating schools, and it was expected that classroom teachers would deliver the program in most instances, only classroom teachers were involved in the research. Forty classroom teachers across the five schools consented for their class to take part. Teacher consent permitted all students in the class to participate in the program as part of their school routine (*N* = 340 students across 40 classes). The number of students in each participating class ranged from 4 to 12, with classes most commonly containing nine students. The students were aged approximately 5–12 years and commonly experienced more than one condition. For example, autism and intellectual disability was frequently reported among children involved in the broader research.

A total of 22 classroom teachers (*n* = 6 males, *n* = 16 females; years worked as a teacher *M* = 13.61) provided feasibility and acceptability data to be analysed in the current study (school 1: *n* = 3 teachers; school 2: *n* = 7 teachers; school 3: *n* = 3 teachers; school 4: *n* = 6 teachers; school 5: *n* = 3 teachers).

### Procedures

Ethics approval to conduct this study was given by the Deakin University Human Research Ethics Committee (2018–179) and the Department of Education and Training (DET), Victoria (2018_003791). This program was also supported by School Sport Victoria, a body governed by DET that provides school sport programs. All methods were carried out in accordance with the Declaration of Helsinki. To recruit schools throughout February–June 2019, details of specialist schools within 80 km of the university with at least 100 students enrolled were collected. Eligible schools were compiled into a randomly ordered list to be contacted via phone and email. To increase recruitment rates, the distance and minimum enrolment criteria were extended after approaching the first round of schools. Thirty-four schools were contacted in total. Other recruitment methods included presenting the program at a specialist school principals’ meeting. Schools specifically for children with physical disabilities and Special Developmental Schools (a type of specialist school that generally caters for students with moderate to severe intellectual disability [18]) were not included in the initial list to be contacted by researchers, as it was believed that the program may require further adaptation before being appropriate for these schools. However, if principals of these schools expressed interest after hearing about the program, they were eligible to enrol.

A researcher (CE) visited interested schools to present the program, either firstly to only leadership staff or directly to all teachers, at the discretion of the school. Ultimately, the program was presented to classroom teachers who were provided with a plain language statement as an invitation to enrol themself and their class. Interested teachers provided written informed consent, consistent with the Declaration of Helsinki. For the purposes of this paper, teachers completed an online survey approximately 4–6 weeks after implementing the program. The same teachers were also invited to complete a semi-structured telephone interview during the 4–6-week period. Telephone, rather than face-to-face, interviews were conducted for feasibility reasons. These evaluations were part of a larger data collection process involving parents and children beyond the scope of this study (see the trial registration for details ACTRN12619000193178). Survey data were collected and managed using REDCap electronic data capture tools hosted at Deakin University [47, 48].

### Intervention

The AJoM program is a classroom-based psychoeducational PA break program developed by researchers in consultation with psychologists, teachers/educators, principals, disability experts and PA/health experts. It is based on a previously evaluated Joy of Moving (JoM) program developed by Pesce and colleagues (e.g., see [3, 49, 50]), which promotes whole child development through designed PA games in an enriched PE program. Modifications to the original JoM program were made, including adopting a brief classroom active break design (and consequently employing different PA games) and incorporating a unique method of fostering competencies in the ‘life skills’ domain of whole child development, particularly emotional regulation. That is, while children’s emotion regulation development (e.g., managing negative emotions) was originally embedded in PA games in the JoM program, in the AJoM program, psychoeducational storybooks are delivered to introduce the notion that PA can be a useful strategy when experiencing negative emotions. The AJoM program has two core ideologies. First, ‘Any movement is good movement’, which also relates to the original JoM approach where children’s exploration and divergent discovery of ways to perform activities is encouraged, and second, ‘Moving helps us feel good’. Thus, AJoM aims to promote children’s enjoyment of any PA while encouraging realisation of the connection between PA and psychological wellbeing (i.e., encouraging a ‘mind-body’ connection). The dual-component AJoM program is implemented for 10-min, four days per week for eight consecutive weeks of a school term. It is administered by classroom teachers in the form of an active break during their general curriculum time and designed to be an addition to existing programs (e.g., PE classes) and break periods (e.g., recess), rather than replacing current PA opportunities.

The first component involves providing psychoeducation using brief (12 page) picture storybooks developed specifically for this program based on the cognitive behavioural model [51]. Accordingly, a connection between thoughts, emotions and behaviour is demonstrated by portraying PA as a strategy to address or regulate emotions. There are eight similar storybooks, one for each week of the program. Seven correspond to emotions (i.e., angry, annoyed, bored, disappointed, sad, scared, worried) and the eighth emphasises inclusivity in PA (i.e., that everyone can move in their own way). Teachers are asked to read the same storybook twice per week at a minimum. The second component involves the class engaging in a movement activity of the teacher’s choice for approx. 8–10 min, four times per week. The movement component adopts a play-based approach centred on the concept of ‘deliberate play’ described in Pesce et al.’s [52] JoM educational program and originally proposed by Côté and Hay [53]. That is, teachers were encouraged to implement activities that were regulated but also allowed children to explore how to play, with the underlying purpose of having fun. Teachers were provided with several activity cards; some contained well-known activities (e.g., Simon Says, Bobs and Statues), while others were adapted from classroom active break resources available online (e.g., ‘Energizers’ [54, 55])*,* or from the original JoM collection [52]*.* While some activities could include academic concepts, these movement activities were intended to be delivered separately to academic learning, in line with the active break design. Each activity card contained possible modifications to extend or simplify the activity and was broadly categorised to assist teachers in choosing an appropriate activity at the time. Categories included ‘cheerful’ (e.g., dance sequences and playful games), ‘calm’ (e.g., stretching), ‘confident’ (e.g., sport inspired activities) and ‘connected’ activities (i.e., require collaboration between students). To allow further flexibility, teachers could opt to implement other movement activities previously used with the class (if applicable) but were asked to ensure that activities met the AJoM principles of getting children moving, being fun, non-competitive and classroom-based (although teachers could take the students outdoors).

Teachers attended a training session (approx. 20 mins) presented by the first author (a doctoral [psychology] student, who was trained and supervised by a team that included clinical psychologists with extensive experience in training and in educational settings for children with disabilities) at their school prior to beginning the program. During the training, teachers were given an AJoM resource kit containing the storybooks, activity cards, a program manual and a logbook (to capture the way the AJoM program was implemented in their class). All resources were also available online. Teachers were trained in the program ideologies and objectives, administration of materials and possible inclusive strategies to get children involved. Presentation slides covering the training content were provided to a leading teacher if there were classroom teachers who could not attend the session.

### Measures

Feasibility and acceptability were assessed using mixed-methods sources collected after teachers implemented the program; see Table 1 for a description. Feasibility in the current study largely considers whether future use of the AJoM program is viable, for example, whether teachers perceive the program to be doable, relevant and sustainable, whether there is demand for the program, whether it is practical and whether it can be implemented and integrated sufficiently in the classroom [56]. Acceptability considers participants’ reactions to the AJoM program, such as the program’s perceived appropriateness, whether there are intentions to continue using it and participants’ satisfaction [﻿^56^]. The investigation of feasibility and acceptability and the outcomes to evaluate each component were guided by five ‘areas of focus’ and associated ‘outcomes of interest’ in a feasibility design framework proposed by Bowen and colleagues [﻿^56^] and previously used by Mazzoli et al. [38]. Our ‘feasibility’ concept encompasses the demand, implementation, practicality and integration ‘areas of focus’ of the framework. Our ‘acceptability’ concept encompasses the framework’s acceptability component only [﻿^56^].

**Table 1:** Mixed Methods Measures of Program Feasibility and Acceptability

Source	Content	Measurement
Teacher post-program online survey	Eight quantitative items adapted from the Toybox-study [57, 58] regarding teacher perception of the AJoM program and experience with implementation (see Fig. 2 for the items).	A 5-point scale from ‘*strongly disagree’* to ‘*strongly agree’.*
	Six custom open-ended (qualitative) survey questions regarding implementation barriers, strengths/weaknesses of the AJoM program, likelihood of continued use, activities commonly used, suggested improvements and other currently operating programs.	Written response
Teacher semi-structured phone interview (post-program)	Seven interview questions: How did you find using the program with your class? Do you see any difference in the way you interact with your students? Are there any aspects of the program that you preferred to use? Did the program stimulate any discussion about mind-body connection? Overall, did you find the program useful? In what way? Can you imagine integrating this program in your teaching practice? Do you have any suggestions for improvements to the program?	Interview transcript

### Data analysis

Quantitative data were analysed using descriptive statistics to evaluate aspects of feasibility and acceptability. Analyses were conducted using IBM SPSS Statistics (version 26). Qualitative analysis began with interview recordings being transcribed by a professional transcription service. Transcripts were checked for accuracy by the first author and uploaded to NVivo 12 Plus (QSR International) for analysis. Two researchers deductively coded the transcripts using key concepts from the definitions of feasibility and acceptability developed for this study (described above). See Table 2 for a list and description of the feasibility and acceptability codes used. Coding began with two transcripts (29% of the interview data) to check for interrater reliability. Discussions were had regarding discrepant coding until interrater reliability was achieved (Cohen’s kappa > 0.8). Sections of transcripts allocated to the codes were then analysed to identify sub-domains by one coder. This was reviewed by the second coder. Transcripts were also analysed inductively by the two coders to identify additional feasibility-related domains in the data that could not be coded with the existing framework. This resulted in “Suggestions for improvement” becoming an additional domain not addressed by the other ‘areas of focus’. The same coding framework and analytical approach was then applied to the open-ended survey data after responses were exported from the database and uploaded to NVivo 12 Plus. Data from this analysis was used to supplement findings from the interviews. The overall process was undertaken in line with the six thematic analysis steps proposed by Braun and Clarke [59].

**Table 2:** Deductive Codes Used in Qualitative Analysis

Focus Area^a^	Code	Brief Description
**Acceptability Codes**		
Acceptability	Appropriateness	Evidence of the suitability of the program (i.e., its structure and activities) for the specialist school setting and students.
	Satisfaction	Evidence of participant satisfaction (including enjoyment, approval, liking, usefulness etc.) or discontent.
	Intention to continue using AJoM	Evidence of intentions for future use of the program.
**Feasibility Codes**		
Practicality	Practicality	Evidence regarding environmental and time considerations related to conducting the program, and level of disruption caused by doing the program.
Demand	Demand for the program	Evidence of the importance of movement breaks for students and reasons why movement breaks are needed.
	Relevance to current practice	Evidence that the school/class already does activities similar to AJoM.
Integration	Integration degree	Evidence of whether the program fits within the school routine.
	Sustainability	Evidence of still using resources at the time of the interview or survey (i.e., post program completion).
Implementation	Doability	Evidence that activities related to the movement break were or weren’t executed during the program period.
	Implementation degree	Evidence related to *how* the activities were implemented (e.g., easy, difficult, facilitators, barriers to implementation etc.).

^a^ ‘Focus area’ refers to the relevant ‘key area of focus for feasibility studies’ proposed by Bowen et al. [﻿^56^]

## Results

**Fig. 1:**
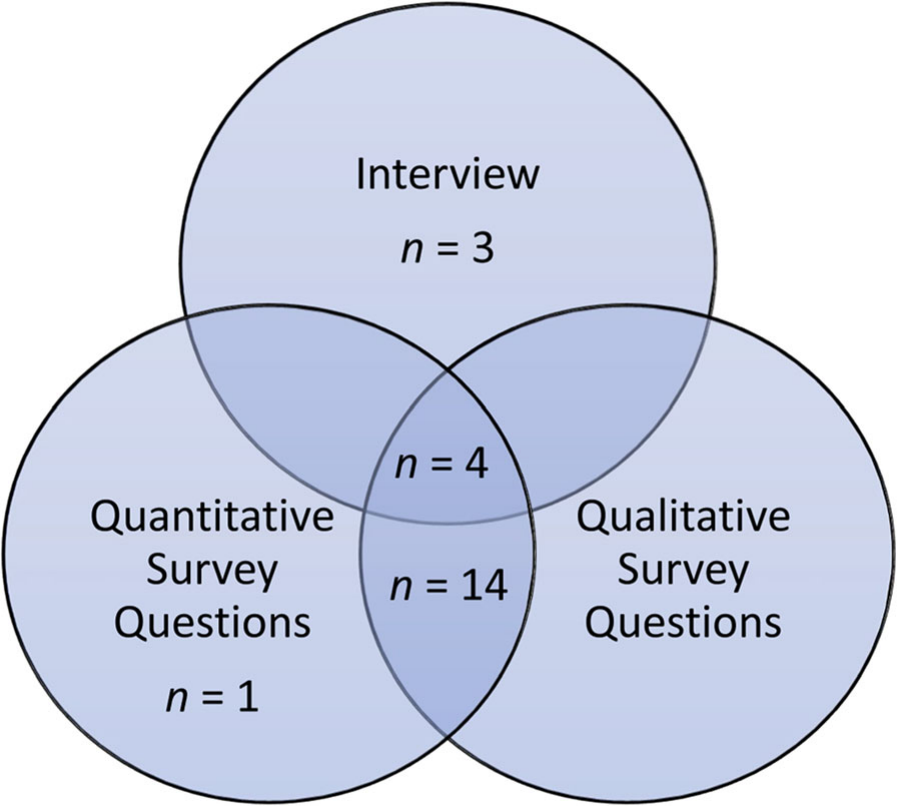
*Venn Diagram Outlining the Number of Teachers that Completed Each Measure in this Study*

**Fig. 2:**
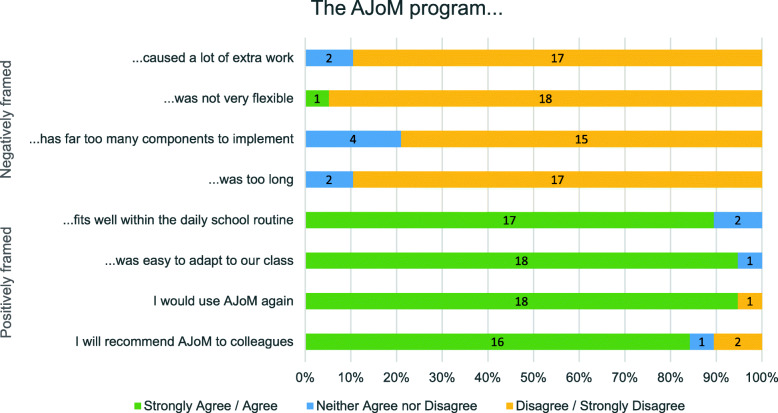
*Quantitative Question Responses Demonstrating Teacher (n = 19) Perceptions of the AJoM Program*

Of the 22 teachers that provided data, seven completed the semi-structured interview, 18 provided qualitative survey responses regarding program perception and 19 completed quantitative program perception questions. Four teachers completed all measures, including both qualitative sources (i.e., the interview and qualitative survey questions), results of which are reported together. See Fig. 1 for a visual overview of the number of teachers that completed each measure or combination of measures. Results of the quantitative program perception questions demonstrate that the AJoM program was perceived positively (see Fig. 2). For a deeper exploration into teachers’ perceptions of the AJoM program, 21 unique teachers were either involved in the semi-structured interview or completed qualitative survey questions. Findings from these sources are combined in the detailed qualitative analysis of feasibility and acceptability below. This analysis enables insight into teacher perceptions of each element of the dual-component program, which can help to understand whether the novel AJoM model is useful in implementing classroom-based PA breaks in specialist schools.

### Acceptability

#### Appropriateness

Teachers provided a range of perceptions regarding the appropriateness of the program’s (a) design and structure, (b) storybooks, and (c) movement activities, as explored below.

##### Appropriateness of the program design and structure

Most teachers interviewed (*n* = 6) and a few survey respondents (*n* = 3) indicated aspects of the AJoM design and structure that were appropriate for specialist primary/junior school classrooms. Some found the organisation of AJoM PA breaks (i.e., the storybook and the movement activities) helpful and considered the “*… strong structure for movement breaks …*” a program strength (Survey-Teacher-10 [S-T-10]). The repetitive aspect (particularly in the storybooks) was also considered appropriate and important to help the students understand the concepts: *“the repetitive nature of the books was great - kids liked the familiarity, and reinforced their learning (especially about endorphins)”* (S-T-2). Some further noted that their students recited the repetitive elements of the books, *“… the kids were almost ringing it off themselves …”* (Interview-Teacher-4 [I-T-4]).

However, a couple of interviewees (*n* = 2) and a few survey respondents (*n* = 4) indicated areas of the program design that were less appropriate for their class. For example, it was noted that while the repetition within the books is good, repeating the same book multiple times per week would not be effective for an upper-primary class (i.e., students aged approximately 10–12 years), suggesting the current design may better suit junior-primary students (i.e., students aged approximately 5–9 years). Similarly, S-T-13 said that a student *“… became bored with the repetitiveness after the second book”*. More generally, some teachers showed concern for the appropriateness of the program for students with higher needs and those that do not engage in regular programs. For example, I-T-5 said *“… I think there could be room for some consultation with like, a special developmental education person …”* and S-T-13 said *“It does not cover all abilities.”*. Taken together, this suggests that while fitting for some groups, aspects of the program design may require adaptation to suit a wider range of classes and specific needs.

##### Appropriateness of the storybooks

Several teachers interviewed (*n* = 4) and a few survey respondents (*n* = 3) indicated appropriateness of the storybooks for their students. For example, S-T-7 commented, “*I loved the books I thought they were a perfect level for all of primary really.”*. Other teachers indicated that the stories seem to be at an appropriate level for students, as students were able to read the books themselves. Furthermore, I-T-1 indicated that the storybook activity was an appropriate task for their class to do and for the students to engage with, as *“… they [students] all sit and listen to the stories, and they love listening to that book.”*.

However, it was suggested that the storybooks may not be suitable for all students. Three teachers suggested that they could be more detailed for upper-primary students: “*The stories for the year 5/6’s were tolerated but were a bit below them. They could have handled a more complex story about needing to move for different reasons.”* (S-T-6). Conversely, two teachers indicated that young students and those with complex needs may have had difficulty comprehending the story, with one suggesting to have *“… a quite simplified version of the story to use with kids that have complex learning needs and complex communication needs.”* (I-T-5). It was also suggested that linking the books to currently utilised curriculums (e.g., the Zones of Regulation, which is a framework that helps students think about their feelings, and gain strategies for self-regulation and emotional control [60]) may improve understanding. One teacher further indicated that the mode of psychoeducation (i.e., storybooks) may not be appropriate for students to engage with, as the class do not often sit as a group for activities. Thus, it appears that the appropriateness of the AJoM storybooks is associated with established classroom practices, class dynamics and learning readiness, where they currently seem most appropriate for classes familiar with activities such us group ‘mat time’. Variations of each book and flexibility in the mode of delivery may be required to meet the needs of a wider range of classes.

##### Appropriateness of movement activities

About half of the teachers (*n* = 11) mentioned a variety of factors that suggest the movement activities are appropriate. One teacher indicated that the activities were familiar to the students. Other teachers described particular activities provided in the AJoM program that they frequently used and why they were suitable for the class (e.g., they were simple games, easy to learn, provided students with appropriate instruction, or conversely, less structured activities were more engaging). More generally, a teacher mentioned that *“… the exercises are suitable for a variety of age groups.”* (S-T-6), while another stated “*There wasn’t that many that we had to kind of go, ‘No, that’s not going to work.’”* (I-T-7).

Nevertheless, despite describing activities that were used, nine participants (in *n* = 2 interviews and *n* = 8 survey responses) also suggested that certain movement activities provided in AJoM may not be suitable for all students. Some teachers indicated that this was to do with age, *“Some of the suggested ideas in the [A]JoM package were too advanced for our young students.”* (S-T-1). Another teacher described finding extra resources (e.g., music videos) to pair with the movement activities when the general instruction/modelling suggested on the activity card may not have been sufficiently engaging for students. Furthermore, despite acknowledging that several activities did work, I-T-7 explained that *“There were some of the activities that weren’t really relevant for my classroom or my school setting,”* due to the activity complexity and students’ needs. This was also noted in the survey responses, with teachers commenting that activities were not suitable for some student’s abilities. Reasons included that some activities were too complicated, required too much memory power or were not suitable for the children’s physical abilities. Thus, while most teachers were able to find appropriate activities to use with their students, the development of additional resources and provision of further adaptation suggestions may allow the program to offer extra tasks that connect more meaningfully to more students.

#### Satisfaction

Teachers demonstrated a range of views, from high satisfaction with the overall AJoM program to low satisfaction. Teachers also noted satisfaction with specific elements of the program and ‘conditional’ satisfaction, described below.

##### High satisfaction

Ten teachers (across *n* = 6 interviews and *n* = 5 surveys) demonstrated approval or fondness of the overall program. This was noted from the teacher perspective, *“A very well thought out and planned program.”* (S-T-18), and child perspective, *“My class loved it.”* (I-T-2). Teachers also expressed the usefulness of the program, and an overall sense of appreciation, *“We thank everyone involved with this program for thinking of this and making a difference to these kids and for their lives.”* (I-T-4).

##### Low satisfaction

In addition to demonstrating satisfaction, a couple of teachers interviewed (*n* = 2) and a few survey respondents (*n* = 3) demonstrated some dissatisfaction. Furthermore, an additional survey respondent mostly expressed discontent. Dissatisfaction may have been related to implementation expectations not aligning with outcomes, *“I think the transfer of the message both in instructions and the stories was not as well received as I thought it might be.”* (I-T-5). Discontent also related to students lack of enjoyment of some elements, for example, *“Students found some [of] the activities a bit boring and would ask for a different activity.”* (S-T-4), and the lack of suitability for all students.

##### Specific and conditional satisfaction

Seventeen teachers (across all interviews and *n* = 14 surveys) demonstrated satisfaction with a specific aspect of the program, such as the design or a particular resource. Additionally, four interviewees and a survey respondent suggested that further approval may depend on certain circumstances or conditions, such as the type of teacher, how the resources are used, time factors or the type of class. For example, *“Some of the other activities were a little too difficult for my students to understand or follow but in saying that, fabulous for other classes!”* (S-T-18). See Additional file 1 for further quotes that exemplify these ideas.

#### Intention to continue using AJoM

Twelve teachers (across *n* = 5 interviews and *n* = 10 surveys) demonstrated intent to continue using the program when asked. For example, S-T-9 wrote: *“Would love to continue to use it as a regular part of classroom activities”*. Teachers also demonstrated interest in either (a) using additions to the program in the future, if the program is extended, *“Happy to do anything in the future that you propose or plan.”* (I-T-4), or (b) using the program with a new class in the future, *“… I plan on using the books next year with my new class as a program and trying it all again.”* (I-T-2). However, in six survey responses, teachers explained that they were either uncertain whether they would continue to use the program, *“I am unsure, it would depend on the nature of the class and their understanding levels.”* (S-T-5), or indicated that they do not intend to deliver the full program (e.g., will only use particular elements of the program or only implement it once per week).

Overall, while it is evident that there are components of the program that require adaptation to be acceptable for more classes, the variety of positive reactions from teachers demonstrate an element of acceptability of the AJoM program.

### Feasibility

#### Practicality

While S-T-14 commented that *“The short time element required is perfect and does not encroach on the children’s valuable learning time”*, three interviewed teachers reported time difficulties related to delivering the program, such as being time poor. I-T-5 suggested that the actual implementation may be time consuming*, “… to use it for a movement break, kind of bringing them together to read the book is quite a bit of time in itself, and then to go out and do the activity.”*. Additionally, a survey respondent reported that time constraints were a challenge, explaining that it was difficult to implement the program on days where students attended other specialist programs. Furthermore, two teachers indicated that delivering the program in its current form may be disruptive for their class. For example, S-T-7 said, *“When it was time they would leave (abscond) so I would only have 1 or 2 students.”*. Notably, no teachers raised space/environment constraints as an issue.

#### Demand

##### Demand for the program

Teachers demonstrated demand for the program by describing the importance of (a) movement breaks and (b) emotional learning for their class.

##### ***Importance of movement***

A few teachers (*n* = 3) expressed that students need movement or movement is important for them. For example, *“I think, regardless of the setting students need them [movement breaks].”* (I-T-7). One also highlighted the particular importance for children at specialist schools, *“I think the program is vital, to be quite honest, and more so in the special needs area which is where I’m teaching.”* (I-T-4). Four teachers (including the three that expressed the importance of movement) noted a variety of reasons for using movement breaks, including for management reasons, to give students a break and break down lessons to increase engagement, and to accommodate students’ needs, especially for very active students, *“… I’ve got some kids with quite a bit of energy and can’t sit for too long, so the movement and stuff is quite important to us.”* (I-T-5). This importance indicates that some perceived demand for the AJoM program may exist.

##### ***Importance of emotional learning***

Two interviewed teachers also indicated the importance of emotional learning, which may be another feature that generates demand for the AJoM program. One teacher stated *“… I’m always looking for books that have relevance to the feelings.”* (I-T-3), while another mentioned the importance of a social and emotional learning program already provided by the school. A survey respondent further indicated the prominence of emotional learning, commenting that “*The stories on different emotions were really great and students have enjoyed listening to them and we do it quite often as and when required.”* (S-T-4), suggesting that demand for these learnings or conversations exists. The AJoM program may therefore be of interest to specialist schools, as it addresses concepts that appear to be valued by some teachers and important for students.

##### Relevance to current practice

Teachers demonstrated that the AJoM program appears relevant to their current practice by describing existing use of similar activities/programs including (a) movement in the classroom, and (b) other programs related to AJoM concepts, both of which are explored below. This may further suggest teachers’ interest in using the AJoM program, thus possibly indicating demand for the program.

##### ***Previous use of movement in the classroom***

Most interviewed teachers (*n* = 5) commented that they had used movement breaks in the classroom prior to the AJoM program. For example, *“With my class, even before we started Joy of Movement [sic] Program, we did a movement break...”* (I-T-1). This was also seen in two of their survey responses. Interestingly, three teachers noted that their previous movement breaks were either not conducted in an organised or consistent fashion, or contained less variability in the activities than the AJoM breaks. Furthermore, four of the five teachers that had used movement breaks previously indicated that implementing the AJoM program was not too different from, or complements, existing classroom practice. Survey responses were less conducive to reporting previous classroom practice, as teachers were asked about their use of *current* programs (i.e., *after* the AJoM program). While teachers did report use of movement breaks in their survey responses, it is less clear whether these were implemented prior to AJoM. Nonetheless, the previous use of movement reported by several teachers could infer further demand for, and feasibility of, the AJoM program.

##### ***Use of other related programs that suggests interest in AJoM***

Fifteen teachers (across *n* = 4 interviews and *n* = 14 surveys) mentioned other programs being used and areas of focus for schools and teachers that align with key concepts in the AJoM program. These included relaxation/meditation programs, the Zones of Regulation curriculum [60], school emphasis on behaviours and emotions, and active/sports programs (e.g., dance classes, daily run/walk in the school grounds etc.). For example, I-T-3 said *“With our school, I think it’s really, it’s probably more significant because there’s a huge emphasis on behavioural management and we’re always looking for different ways for the children to understand their emotions.”*. This further suggests that the AJoM content may be associated with interests of schools and teachers, indicating that the program could be feasible for use in the specialist school setting and perhaps even likely to be used.

Thus, conceptually, the AJoM program seems relevant to specialist schools, with the core concepts and goals appearing to fit the culture of schools. Demand for the AJoM program may therefore exist, given the importance and considerable prior use of movement breaks, and the value of learning emotional and other aligned concepts. However, it is important to consider that, as previously mentioned in ‘*appropriateness’,* there may be instances where the program’s methodology is less aligned with established teaching practices in some classes.

#### Integration

##### Integration degree

While the above narrative suggests that the AJoM program may integrate into classrooms, as teachers’ evidence considerable prior use of movement breaks, the below demonstrates where and how teachers may fit the program into the school day or class routine. When asked specifically about the possibility of integrating the program into ongoing teaching practice, several interviewed teachers (*n* = 5) believed that they would or could imagine integrating the program.

##### ***Potential to fit class schedules***

Half the teachers (*n* = 11) provided evidence of the program potentially integrating in their class schedule. Some indicated that movement breaks can fit their routine by highlighting the number of times their class does them per day, *“Our class typically has 2-3 timetabled movement breaks per day with changes depending on student need.”* (S-T-5). Some teachers specifically reported *where* and *how* movement activities fit their schedule by describing where movement breaks are positioned in their timetable, *“10min active breaks within the classroom in the morning sessions …”* (S-T-3). Others reported using movement if students are not attentive, have been sitting for a long time or in between lessons, *“… usually we have two activities together, like reading and writing, so what we do is, when we finish reading, then we give them a 10 minute break, a movement break.”* (I-T-1). While teachers did not state that each of these instances were specific to AJoM implementation or involved implementing the program as a whole (i.e., movement combined with storybooks), it demonstrates instances where movement breaks could fit with class schedules, which may also be able to include the AJoM storybooks on selected occasions. Indeed, when describing their previous use of movement breaks, I-T-7 said, *“We still had them at the same time as what we did when we were doing The Joy of Moving.”*, indicating that AJoM breaks were able to fit into existing movement break periods.

Some teachers (*n* = 6 of the 11 noted above) did describe the AJoM resources being used, demonstrating possible integration specific to AJoM. For example, *“And I was doing that twice a week – reading the book twice a week and doing the program almost every day …”* (I-T-6). Similarly, I-T-1 reported that the program became part of their timetable, and S-T-14 wrote *“… we have an action-packed curriculum, but we can always find the 5-10 minutes required to implement this program.”*. Interestingly, a teacher also described the program being used with individual students in response to their needs. For example, during the study period, if a student felt frustrated, they would read an AJoM storybook and then do a movement activity until they felt ready to re-join the class. While not necessarily intended to be used individually, this exemplifies another way in which teachers may integrate the program for meaningful use in this setting. The above evidence, along with some teachers noting that the program was not too different from their current classroom practice (as previously discussed), may indicate that the AJoM program could integrate sufficiently into class schedules.

##### ***Integration challenges***

Several teachers (*n* = 8) also described factors that may hinder successful integration into the school structure. These included time factors (mentioned previously in ‘*practicality’*) and specific class practices (i.e., not often sitting together as a group, as noted previously in *‘appropriateness’*). Additionally, S-T-3 reported experiencing difficulty introducing a new program into their existing class routine, *“The most challenging aspect was changing my morning routine to fit in the Joy of Movement [sic] program with my class who are highly autistic and like their set daily routine.”*. Other difficulties related to staffing, where the absence of school leaders or classroom teachers could create integration challenges by impacting areas of the general curriculum (making it difficult to integrate additional programs) or routine. While this is not amendable or specific to the AJoM program, it does present a consideration for ensuring consistent integration of the program. Relatedly, S-T-7 explained that consistent integration was not successful, due to placing emphasis on other areas of the students learning, *“Towards the end of term it dropped off in class as honestly we were to [sic] busy focusing on behaviours.”*.

##### Sustainability

Four teachers provided evidence of still using the AJoM activities during their interview (i.e., after program completion), indicating sustainability of the program. This was demonstrated for the overall program, *“And even if the thing is over we’re still doing it …”* (I-T-1), as well as specific aspects of the program, such as movement, *“I already still like to do a 5-10-minute activity, moving activity during the day with the kids …”* (I-T-2), or storybooks, *“We have continued to use the stories in our movement break …”* (I-T-7). Just one teacher explicitly reported an aspect that was not being used anymore *“I’m not using the books anymore. I’m just using the … just, you know, just talking them through it verbally …”* (I-T-2). This may suggest that while the tasks themselves are sustainable, the AJoM program may require additional resources (particularly storybooks, to avoid repeating the same eight) if it is to be used by teachers long-term. Sustainability was not mentioned by teachers in their survey responses, and it is not possible to know whether the remaining interviewed teachers were still using program resources. Nevertheless, the element of sustainability portrayed supports the ability to integrate the program into the school system.

Taken together, although not without challenges, there is evidence to support being able to integrate classroom-based PA breaks in the school routine. However, further research would be useful to better gauge the ability of more teachers to integrate the AJoM activities, particularly in relation to adherence to the prescribed dosage.

#### Implementation

##### Doability

All interviewees demonstrated at least one element of the AJoM program (i.e., movement or storybooks) that was able to be done. Additionally, most survey respondents (*n* = 17) provided evidence of activities that were commonly used. Most teachers (*n* = 20) indicated that doing a movement activity was possible by describing the use of either an activity provided in the AJoM package, *“Sometimes it is Simon Says, which they enjoy, or it’s the Chicken Dance …”* (I-T-1), or an external activity, some of which involved unstructured activities, while others commonly involved music, for example, *“… copying movements on YouTube music videos, such as Jack Hartmann’s Count to 100.”* (S-T-2). Several teachers (*n* = 10) also demonstrated that the storybook component was doable. For example, *“Sometimes I read it three times, but it was basically twice, you know. Once I think at the beginning and then another time at the end of the week.”.* (I-T-4).

However, seven teachers also reported being restricted in their implementation of some activities. Most instances were related to elements previously discussed as lacking appropriateness. For example, some movement activities provided in the AJoM package were not able to be done by all classes, with one teacher explaining that they *“… did not use any activities from AJoM.”* (S-T-13), as many were not applicable to the students’ abilities. While teachers are not required to use every (or any) activity provided and are permitted to use their own activities, revising some activities could provide classes with more options and increase the accessibility of the program. Additionally, of note, the program was not always done as intended and therefore may have deviated from the program aims. For example, one teacher reported that it was *“… used as a literacy tool as opposed to, you know, movement and dance and addressing the emotions.”* (I-T-3).

#### Implementation degree

##### ***Ease of implementation***

Several interviewees (*n* = 5) indicated that the program was easy to implement and identified facilitators of implementation. Facilitators included the program being relatively simple, *“The program’s pretty straight forward.”* (I-T-7), the teacher being familiar with movement breaks, *“Oh it was very easy to implement for me because I’m doing that anyway”* (I-T-4) and having variety in the activities, *“… you could easily find activities that suited everyone. And most of the activities you could easily scale up or scale down, if needed.”* (I-T-7). Elements that were easy or simple to implement were also identified in the survey responses (*n* = 5), with most focussing on specific movement activities. For example, in describing commonly used activities, S-T-18 said *“We repeated these activities quite a lot as they were familiar and easy to follow. My students struggle with too many instructions or ‘rules’ so it was nice to keep it simple.”*.

##### ***Additional implementation considerations***

Four interviewed teachers mentioned additional factors, including resources and teacher attitudes, which may influence the way the program is implemented. Regarding resources, I-T-1 mentioned support staff being involved with implementing the program, although this appears to have been used most when implementing the program with individual students. Additionally, as indicated previously, teachers mentioned frequently using songs or music videos in their implementation of the program, for example:

… *I was able to find kind of like, a letter, or like a music video kind of thing, where it modelled someone writing out the letters with their arms or their feet or whatever, and so it kind of gave them [students] something a bit to connect to* … (I-T-5).

This was echoed in several (*n* = 5) survey responses, *“We commonly engaged in action songs, both with or without the support of videos/ songs on YouTube.”* (S-T-1). While not prescribed as requirements of the program, it is worth noting these factors that may facilitate easier implementation or greater student engagement in the program. Similarly, S-T-1 described creating extra resources that were vital to successful implementation and therefore must be considered for future iterations of the program, *“In order to get the program working more effectively, I was required to make up my own class visuals to give students understanding, provide choice and to enable a more predictable routine.”*.

One interviewee commented on the influence that teacher attitudes may have on program implementation, noting:

*If the teacher embraces it, the kids will read that and see that they really enjoy it as well, it’s more likely to be – it will be much more effective and more likely to be done consistently in an enjoyable way.* (I-T-4).

***Challenges to implementation related to program design.*** Despite several teachers noting that the program was easy to implement, four (across *n* = 3 interviews and *n* = 1 survey) also reported aspects of the program design and instruction that created potential implementation barriers. Most related to previously mentioned time challenges, *“… sometimes the 10 minutes seemed a bit long on days when there is a lot of physical activity anyway …”* (S-T-2). Furthermore, despite indicating that the program content specifically was easy to administer, I-T-3 indicated that the program instructions may contribute to overall implementation challenges or confusion. Additionally, I-T-3 said *“But with that said, it was something where, yeah I would have liked to have spent more time with knowing how to roll it out. Because I was just left up to my own devices.”*. This could contribute to the program not being implemented as intended (as described above). Therefore, the training presentation and manual provided may need to be reviewed to ensure instructions are as clear as possible to support teachers.

***Challenges to implementation specific to teachers or the class.*** Again, while various teachers mentioned that the program was easy to implement, just over half (*n* = 12) identified teacher and class specific circumstances that created barriers to implementation. Most related to elements that were previously covered in the discussion of *‘appropriateness’* (e.g., too much repetition of storybooks for an upper-primary class, some movement activities being too difficult for students), suggesting that some areas of implementation may lack feasibility with particular groups. Another challenge was engaging and motivating students. S-T-2 said it was *“difficult to get all students involved if some decided they didn’t want to do it …”* . Moreover, while not prescribed by the program, I-T-3 suggested that a lack of collaboration with colleagues may be a barrier to successful implementation, *“… with the other, my colleagues, we never discussed what we did.”*.

Overall, while the implementation findings above suggest that the activities involved in the AJoM program can be delivered in several classes, further adaptations to overcome the noted difficulties could increase the success of implementation and increase the accessibility of the program in the classes that had more significant challenges.

#### Suggested improvements

Teachers were also given the opportunity to provide suggestions for improvement to the program in the interview and survey, providing further insight into how they perceive the program.

Three teachers (in *n* = 2 interviews and *n* = 2 surveys) suggested changes related to the movement activities. For example, the program could include resources (e.g., sensory materials) to engage students that are not interested in doing a particular activity chosen for the class, as explained by I-T-1: *“Some children don’t like yoga, when everyone’s doing yoga, but I cannot say that ‘No, we are not going to do this’ so change to something else. Maybe engage those kind of students with some other material …”* . The inclusion of ‘hands-on’ and sensory activities may also engage children with a wider range of physical abilities, as suggested by S-T-10, *“Sensory activities (finger play, manipulating objects, etc.) perhaps to some of our less active (due to physical limitations) students.”*.

Several interviewees (*n* = 4) and a couple of survey respondents (*n* = 2) suggested changes related to the psychoeducation component. This included creating a video version of the storybooks and designing more books, *“Maybe a variety of more books, because those books are really beautiful …”* (I-T-1), which in fact indicates a positive perception of the program. Other areas for improvement were previously mentioned in the discussion of *‘appropriateness’* (i.e., reduce repetitiveness and create more detailed and simplified versions of the books).

A few interviewed teachers (*n* = 3) and survey respondents (*n* = 5) suggested changes related to the program design/set-up, some of which were previously identified as implementation considerations and challenges (i.e., remove the time requirement, review instructions and paperwork for clarity, and include visuals to help students with routine and communication). Additionally, although consultation with specialist school staff was undertaken during program development, a teacher indicated that further consultation may be necessary to increase appropriateness for more students. Other suggestions included introducing indigenous elements, having an optional participation tracking chart for students, matching movement activities with the storybooks and demonstrating an AJoM session prior to teachers beginning implementation, *“Maybe a demonstration with the class so that the standard is set and all teachers are under the same impression of what is expected.”* (S-T-18).

Notably, some teachers who suggested improvements also expressed positive perceptions about the program. For example, I-T-6 said *“I think it’s perfect. It’s wonderful if people can do it.”* but went on to suggest removing the time requirement. Additionally, five teachers indicated that there were no changes they would make, demonstrating satisfaction with the program’s current form. Five survey respondents did not answer this question.

## Discussion

The aim of this pilot study was to investigate the feasibility and acceptability of implementing a classroom-based PA break program in Australian specialist primary/junior schools. Using a novel program (the AJoM program), this study demonstrates preliminary evidence for the feasibility and acceptability of implementing classroom-based psychoeducational active breaks in several specialist classes. However, variation exists between teachers’ perceptions, with common divergences in perceptions appearing to relate to the age and developmental level or needs of the class. This demonstrates the importance of allowing extensive flexibility in classroom-based PA break programs.

### Acceptability

A variety of positive perceptions indicate there is evidence for a degree of acceptability of the AJoM program. Most teachers expressed satisfaction with at least one element of the program, reported that they would recommend the program to a colleague and several voiced students’ enjoyment of the activities. Intent to continue using the program was also demonstrated both qualitatively and quantitatively. Some teachers that were unsure if they would continue using the program expressed that this depended on the nature of their class, signifying acceptability in some classes but not others. Appropriateness was also only demonstrated for some classes. This appeared to relate mostly to the age of the students (i.e., less appropriate for some of the youngest and oldest students) or student ability (i.e., less appropriate for students with high needs). This aligns with the only other feasibility study of active breaks in specialist schools to our knowledge, which also found that appropriateness may be related to age [38]. Although their study explored cognitively challenging active breaks, Mazzoli et al. [38] found that the tasks were more appropriate for children of later chronological age but developmental age of 6–8 years. Importantly, most teachers that completed the quantitative program perception questions in the current study agreed that the AJoM program ‘was easy to adapt to our class’. Therefore, while some modifications would be useful, it appears that even in the program’s current form, many teachers were able to tailor the program appropriately to their class.

While some challenges to acceptability identified in this study (i.e., age appropriateness of activities) have been previously acknowledged as factors to consider in implementing school-based PA in both specialist and mainstream schools [38, 61], other factors, such as challenges related to the appropriateness of the tasks for students’ abilities, may be more relevant to specialist schools [39]. This might be explained by the notion that the optimal challenge point in PA differs according to children’s motor development level, as children with motor difficulties may require higher executive control to perform motor tasks than those without [62]. Given the heterogeneity of students at specialist schools, it seems important to identify and accommodate various optimal challenge thresholds, perhaps at the class level, to ensure a balance between task difficulty and ability [38, 62]. This relates not only to the movement tasks but also storybooks to increase the appropriateness of classroom-based psychoeducational PA breaks. Results of the current study indicate that it may also be relevant to consider appropriateness with respect to the level of repetition of activities. While repetition may assist in optimising learning in some classes, in others, it could diminish perceived variety, which has been shown to predict autonomous motivation [63].

### Feasibility

Findings also demonstrate preliminary feasibility of the program. No teachers expressed that the AJoM concepts are irrelevant to their students. In fact, many reported prior use of programs containing similar concepts, demonstrating potential demand for, and the utility of, AJoM in these classrooms. Additionally, the program appears to be practical for some teachers to implement, as none that completed the quantitative program perception questions agreed that it ‘was too long’ and no environmental/space issues were discussed. However, time constraint challenges were described qualitatively, challenging feasibility for some teachers. Time also appeared to challenge the ability of some teachers to integrate the PA breaks, along with other factors (e.g., staff absence and specific class characteristics). Nevertheless, integration was feasible in some instances, with a few teachers demonstrating how they integrate sessions into their routine and others demonstrating sustainable use. Furthermore, no teachers disagreed that the AJoM program ‘fits well within the daily school routine’. Notwithstanding challenges, findings also show that it is possible for several teachers to sufficiently implement the program. This was seen qualitatively, where some teachers indicated that the program or activities were easy to implement. Quantitatively, no teachers agreed that the program ‘has far too many components to implement’ or ‘caused a lot of extra work’, suggesting that implementation was not overly complex. However, there were barriers to implementation and elements of the program that were not able to be done in some classes, indicating that modifications would be useful.

Some challenges to feasibility identified in this study have been previously acknowledged as potential barriers to school-based PA. These include time constraints, lack of student engagement/motivation and disrupting or unsettling the class environment [28, 36, 61, 64–66]. However, it is worth acknowledging differences in implementation between mainstream and specialist schools that may contribute to feasibility. While previous literature suggests that activities requiring few materials and minimal preparation are generally preferred by teachers [36, 66], the teachers in our study didn’t seem concerned about using additional resources (e.g., songs, videos) in their activities. This aligns with findings of McMinn and colleagues [39], where several of the classroom PA tasks judged as most suitable for children with special educational needs included music or song. Interestingly, in a study by Stylianou et al. [67] where classroom-based PA resources were provided in several modalities, teachers preferred a packet of activity ideas over DVDs and links to online resources. Whereas in our study, teachers often chose to use songs and online videos as well as, or instead of, the activity instruction cards provided that largely required no equipment. Only one special education teacher was included in the study by Stylianou et al. [67]. This supports the idea that incorporating PA in classrooms would differ between mainstream and specialist schools, and may relate to the utility of visual supports for children with disabilities [45]. Indeed, the use of visual aids such as storyboards and routine boards contributed to the feasibility of implementing a 30-min school-based exercise intervention for children with moderate-to-severe intellectual disability in a study by Bellamy et al. [26]. Thus, perhaps the time and effort expended setting-up visual resources (e.g., online music videos and routine boards) is worthwhile to increase student engagement in specialist classes.

### Recommendations

Overall, findings demonstrate that while there is evidence for the classroom-based psychoeducational PA program to be feasible and acceptable in specialist schools, variation exists in teachers’ experiences. Thus, the primary recommendation of this research is that a flexible approach to design and implementation of classroom-based PA breaks that puts choice and variety at the core of the program be adopted to allow for highly tailored classroom PA. Other important recommendations emerging from this study that should be considered in designing and implementing future classroom-based active break programs in specialist schools include (1) program design must include extensive collaboration with special education teachers and experts, (2) visual aids (e.g., storyboards) could be provided to assist classes with adjusting and understanding the routine, (3) it would be useful to build demonstrations into teacher training sessions and ensure instruction materials are as clear as possible to further support teachers.

In line with the primary recommendation, the importance of flexibility in the delivery and level of tasks was a recurring idea identified in the results of this study across the AJoM intervention components. This confirms what we would expect in a setting with considerable heterogeneity at both the student and class levels [41]. This recommendation would provide the flexibility required to coincide with the complexity of specialist schools from two angles, (1) empowering teachers to decide how to best adapt the program to meet the needs of their class in terms of dose, how, and when the program is implemented, and (2) providing a large variety of resources in differing formats to cater to varying ages and needs of the children. This recommendation aligns with the UDL framework, which encourages the use of flexibility in classrooms to provide appropriate accommodations and supports for all students [68]. Indeed, the UDL framework recommends using multimodal methods to present material, engage children with material and allow children to demonstrate skills [68]. This approach also supports opportunities to accommodate differing optimal challenge points for children at specialist schools. Some examples of applying this flexibility in practice could include (1) ensuring movement activities have ample modification options as well as a wide variety of tasks, including object manipulation, (2) providing supplementary resources that appear to be commonly used with movement activities (e.g., songs or video demonstrations of each activity) on a host website, (3) in the AJoM program specifically, allowing teachers to choose between providing psychoeducation content using a simplified animation video (to possibly assist with engagement where it is difficult to gather for a story) or a more detailed book (to appeal to older students).

Prior literature supports the importance of designing programs that can be adapted to meet the needs of different student populations to encourage effective implementation [36, 69] and indicates that a bespoke approach is required to facilitate PA participation for children with disabilities [70]. This also aligns with findings of Mazzoli et al.’s [38] feasibility study of active breaks in specialist schools, which demonstrate that a cognitively challenging motor task may be feasible if tailored to the specific needs of students. Promisingly, it appears that many teachers in our study were able to apply the elements of flexibility in the AJoM design to tailor components to their class, for example, by choosing activities that engaged students most if some were deemed boring, or using their own movement games if those provided were not suitable. Flexibility was also acknowledged quantitatively, with most teachers disagreeing that the program ‘was not very flexible’. Although the UDL framework is not intended only for children with disabilities, specialist school teachers may be particularly familiar with applying UDL principles and therefore utilising flexibility to adapt programs, given highly individualised planning is characteristic of special education [40]. Nevertheless, the results of this study extend current knowledge to more clearly recommend that flexibility should cover all aspects of a classroom-based active break program (e.g., dose, teacher implementation and all resources).

This recommendation also seems applicable to classroom-based active break programs designed for mainstream schools. Indeed, Watson and colleagues [71] found that flexibility around implementation and usage frequency may be necessary for classroom active breaks in mainstream schools. Given many children with disabilities attend mainstream schools, extensive flexibility in classroom-based active breaks, including flexibility in resources and in delivery options, may encourage more effective participation of all children. However, given previous research has found that teachers often prefer activities with minimal equipment and preparation [36, 66], further research is required to understand whether providing a variety of supportive resource options (e.g., songs, music videos, object manipulation) would be feasible for teachers at mainstream schools. Further research would also be useful to better understand whether the use of these resources impacts feasibility associated with time constraints in specialist schools.

### Strengths, limitations and future research

This study adds to limited knowledge of the feasibility and acceptability of classroom-based PA breaks in specialist schools and investigates a novel program that combines both physical and psychological health constructs in a naturalistic intervention for children with disabilities. However, it is not without limitations. Firstly, given a whole school approach was not required upon enrolment to the study, participating classes may be biased towards teachers that value PA breaks, already conduct them, or thought the program would be suitable for their class after attending the recruitment presentation. This may limit the ability to truly understand feasibility and acceptability. Secondly, a similar bias may be evident in the quantitative program perception questions and semi-structured interviews. Given only 19 teachers completed the quantitative questions and all perceived the program quite positively, it is possible that the teachers with favourable views of PA breaks chose to respond while others did not. Additionally, interview data represent teachers from only two schools. While qualitative survey responses represent all five schools, it would be insightful to have in-depth interview data from teachers at more schools. Thirdly, although schools that cater for students with mild to profound intellectual disability were involved with this study, this did not include any Special Developmental Schools (a type of specialist school that generally caters for students with moderate-to-severe intellectual disability [18]). Further research is required to understand feasibility and acceptability in these settings specifically. Finally, there is limited evidence about adherence to implementation dosage (particularly frequency). While this information was gathered from some teachers during visits to the schools, the information was challenging to collect from most, as researchers were careful to not disrupt teachers during class time. Future research into teachers’ ability to adhere to the program dosage would be useful to clarify this remaining uncertainty about feasibility. Additionally, since activities were used with individual students in some instances and this was not the expected use for the program, this would be a beneficial area for future research to explore further to add to the knowledge base of active break use in specialist schools. Further research is also needed due to the pilot nature of this study.

## Conclusions

This study demonstrates preliminary evidence that conducting classroom-based psychoeducational PA breaks in specialist schools can be feasible and acceptable in several classes. However, extensive flexibility across all aspects of the program is required. Future research should continue to investigate the feasibility and acceptability of classroom-based PA breaks in specialist schools, as well as evaluate outcomes to determine their effectiveness and associated benefits. Despite the limitations and further research required, classroom-based PA breaks may be a viable method of getting children with disabilities more active during the school day.

## Supplementary Information

**Additional file 1.**

## Data Availability

The datasets generated and/or analysed during the current study are not publicly available as not all participants consent to their data being used for purposes other than those described in the original study outline. The availability of the data are subject to the provisions of the ethics application.

## Abbreviations

AJoM: Australian Joy of Moving
DET: Department of Education and Training
JoM: Joy of Moving
MVPA: Moderate-to-vigorous physical activity
PA: Physical activity
PE: Physical Education
UDL: Universal Design for Learning
