# Supplementary material for: The feasibility and acceptability of a classroom-based physical activity program for children attending specialist schools: a mixed-methods pilot study
Source: BMC Public Health. 2022 Jan 6;22:40. doi: 10.1186/s12889-021-11990-4 (PMC8740057; doi:10.1186/s12889-021-11990-4)
Supplement: Supplementary file 1 — Additional file 1. [file 12889_2021_11990_MOESM1_ESM.docx]

**Additional File 1**

*Types of Conditional and Specific Satisfaction with Example Quotes*

| Condition or Program Aspect | Example Quote |
| --- | --- |
| **Conditional Satisfaction** | |
| Type of class | “Some of the other activities were a little too difficult for my students to understand or follow but in saying that, fabulous for other classes!” |
| Type of teacher | “But like I said it’s up to the teacher to really embrace it and enjoy actual movement … So, that type of teacher, yeah, it’s going to be a winner.” |
| How resources are used | “…I’ve integrated that with another unit that’s offered … where they talk about all the different zones and emotions and yeah, I think that’s where it [the AJoM program] was… I found it very effective for the class.” |
| Time factors | “Yeah, yeah, it’s good. The only thing when it’s like, you know, if it has to be done for ten minutes, and, you know, then it takes a lot of time…” |
| **Specific Satisfaction** | |
| Program design/structure | “…[before AJoM] we didn’t have any kind of books or anything, we would just give them a 10 minute break, and it was not in an organised way. And bringing this organisation into the classroom has definitely helped …” |
| Resource- storybooks (teacher perspective) | “The presentation is brilliant, you know, the way the books have been done…” |
| Resource- storybooks (student perspective) | “The students really took to the books and they liked that the books were quite cool and they had repetitive elements to it throughout each book.” |
| Resource- movement tasks (teacher perspective) | “Many different games to implement and they were fun.” |
| Resource- movement tasks (student perspective) | “These [particular activities described by teacher] were simple games that were easy for the kids to understand and fun for them to participate in. We added our own elements to these games which the students liked.” |
| Concepts covered by AJoM | “It was good connecting movement to emotions…” |
